# Seborrheic Keratoses as the First Sign of Bladder Carcinoma: Case Report of Leser-Trélat Sign in a Rare Association with Urinary Tract Cancer

**DOI:** 10.1155/2016/4259190

**Published:** 2016-11-23

**Authors:** Aline Stollmeier, Bernardo Augusto Rosario, Bruna Loise Mayer, Gibran Avelino Frandoloso, Francisco Luiz Gomide Mafra Magalhães, Gustavo Lenci Marques

**Affiliations:** ^1^Federal University of Paraná, Curitiba, PR, Brazil; ^2^Internal Medicine Department, Clinics Hospital of the Federal University of Paraná, Curitiba, PR, Brazil

## Abstract

*Introduction*. Skin disorders can be the first manifestation of occult diseases. The recognition of typical paraneoplastic dermatoses may anticipate the cancer diagnosis and improve its prognosis. Although rarely observed, the sudden appearance and/or rapid increase in number and size of seborrheic keratoses can be associated with malignant neoplasms, known as the sign of Leser-Trélat. The aim of this report is to unveil a case of a patient whose recently erupted seborrheic keratoses led to investigation and consequent diagnosis of bladder cancer.* Case Presentation*. A 67-year-old man was admitted to the intensive care unit due to an exacerbation of chronic obstructive pulmonary disease (COPD). On physical examination, multiple seborrheic keratoses on the back of the hands, elbows, and trunk were observed; the patient had a 4-month history of these lesions yet was asymptomatic. The possibility of Leser-Trélat syndrome justified the investigation for neoplasia, and a bladder carcinoma was detected by CT-scan. The patient denied previous hematuria or any other related symptoms. Many of the lesions regressed during oncologic treatment.* Conclusion*. Despite the critics on the validity of the sign of Leser-Trélat, our patient fulfills the description of the disease, though urinary malignancy is a rare association. That corroborates the need of further investigation when there is a possibility of paraneoplastic manifestation.

## 1. Introduction

Paraneoplastic diseases may be defined as hormonal, neurological, or hematological disturbances and as clinical and biochemical imbalances related to the presence of malignancies, with no direct association with primary tumor invasion or metastasis [1]. If promptly recognized, skin manifestations can lead to an early diagnosis of neoplasia, improving its prognosis [2]. Among the cutaneous findings is the rare and controversial sign of Leser-Trélat, characterized as the sudden appearance and/or increase in number and size of seborrheic keratoses, associated with an internal neoplasm [3].

The aim of this case report is to describe a cancer diagnosis made after following the clue given by the patient's skin condition—a case of Leser-Trélat syndrome.

A written informed consent of the patient has been obtained for the publication of this case report.

## 2. Case Presentation

A 67-year-old man was admitted to the intensive care unit (ICU) due to a second exacerbation of COPD in two weeks. He came from a first aid unit, had already been through orotracheal intubation, and was receiving mechanical ventilation and antibiotics. The patient was a heavy smoker (120 pack-years of tobacco exposure) and started to claim dyspnea to great efforts over the previous few months; he had also lost 8 kg in less than a month. There was no other known comorbid condition in his clinical history. After some days of stabilization in the ICU, the patient was referred to the general internal medicine unit, where another exacerbation took place, along with fever and productive cough, likewise controlled with bronchodilators and antibiotics.

**Figure 1:**
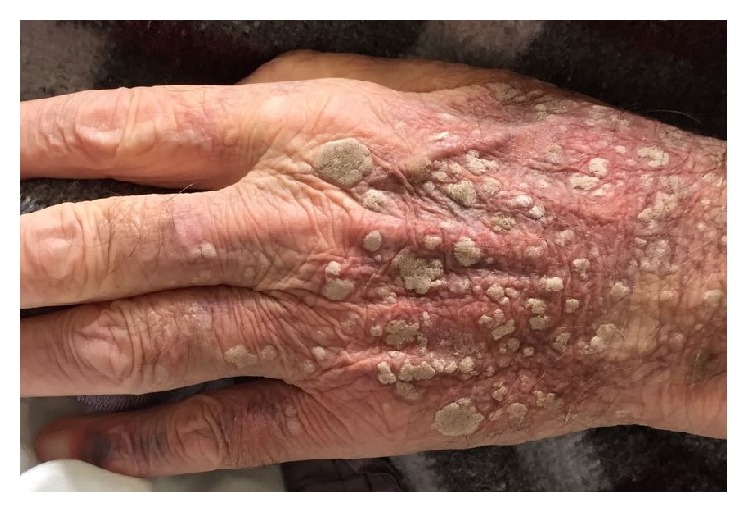
Seborrheic keratoses on the back of the hand.

On physical examination, multiple seborrheic keratoses on the back of the hands (Figure 1), elbows, and trunk were observed. They were asymptomatic and have appeared in the previous four months. These lesions alerted to the possibility of a paraneoplastic manifestation, hypothesis also considered by the dermatologists who followed the case.

**Figure 2:**
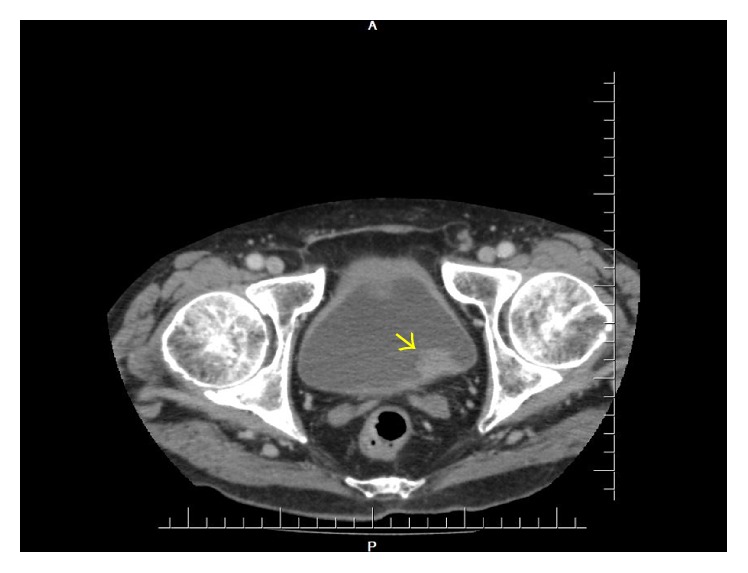
Abdomen CT-scan displaying a vegetation on the posterolateral bladder wall (arrow).

The search for an occult neoplasia was then initiated. The investigation comprehended thorax CT-scan, upper gastrointestinal endoscopy, colonoscopy, and blood analysis, which pointed no malignancy. Colonoscopy showed four sessile polyps, one in the ascending colon (measuring 10 mm), histopathologically defined as a tubulovillous adenoma with high-grade dysplasia, and three in the transverse colon (the biggest measuring 9 mm), corresponding to tubular adenomas with high-grade dysplasia. Ultimately, abdomen CT-scan revealed one irregular-shaped vegetation on the bladder floor, measuring 22 × 18 × 16 mm, with no apparent adipose tissue invasion (Figure 2). The patient denied previous hematuria or any other urinary symptom.

Due to an incomplete stabilization of the pulmonary condition, the patient was discharged and returned after a month for cystoscopy and transurethral resection of the bladder tumor. Histopathological examination of the removed tissue confirmed a noninvasive low grade papillary urothelial carcinoma. Control cystoscopy did not show any residual mass. Adjuvant therapy with intravesical BCG administration was then commenced.

**Figure 3:**
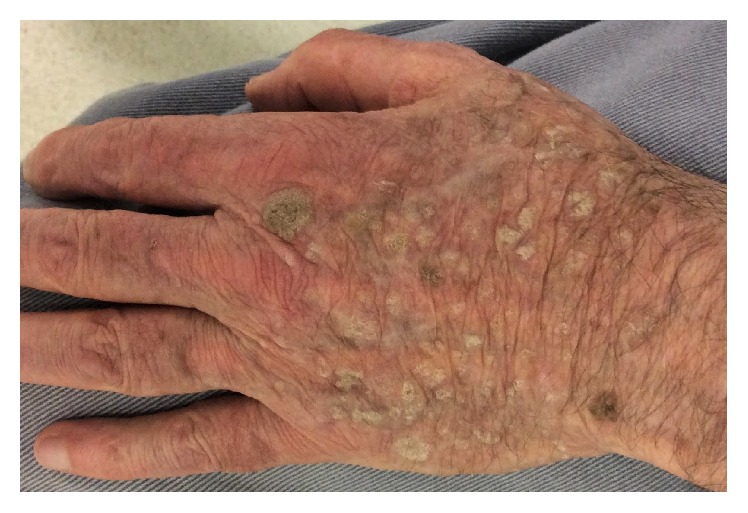
Remaining lesions on the back of the hand nine months after the start of the treatment.

Nine months after the start of the treatment, the patient still presented residual lesions of seborrheic keratoses, which are then pruriginous (Figure 3).

## 3. Discussion

Indirect involvement of the skin by visceral tumors can cause a variety of inflammatory, proliferative, metabolic, and neoplastic changes without the actual presence of tumor cells [4]. In these paraneoplastic disorders, the skin condition generally shows up distant from the primary tumor site. Though mechanisms are unknown in most cases, it is believed that mediators such as growth factors, cytokines, or hormones are involved in the pathogenesis of the cutaneous findings [2].

Six criteria have been described to consider a cutaneous alteration as paraneoplastic, known as Curth's postulates: (1) both conditions (neoplasia and paraneoplasia) are of concurrent onset, (2) both follow a parallel course, (3) the skin lesion is not part of a genetic syndrome, (4) a specific tumor occurs with a certain dermatosis, (5) the dermatosis is not common in general population, and (6) there is a high percentage of the association between both conditions [1, 2, 5]. Not all criteria must be met to demonstrate the link between a skin disease and an underlying malignancy [4].

The sign of Leser-Trélat is defined as the sudden appearance and/or rapid increase in number and size of seborrheic keratoses, secondary to a neoplasia [3, 6, 7]. These are papular, verrucous, usually well-defined lesions of varying colors (brown, black, or tan) which primarily affect thorax and dorsum, followed by extremities, face, abdomen, neck, and axilla [1]. Though the description of the sign has been credited to Edmund Leser and Ulysse Trélat, they were both observing the presence of cherry angiomas in oncological patients, association that probably does not exist; Hollander was indeed the first to document the connection between cancer and eruptive seborrheic keratoses [7, 8].

In 1988, Holdiness gathered the available data (60 cases) to analyze clinical features of the sign. The average age at the time of reported onset was 60.7 years. The average time of evolution of the cutaneous lesions was 14.9 weeks, highlighting its eruptive presentation as a requisite that get most of the patients to be aware of. Acanthosis nigricans and pruritus were simultaneously present in 16.7%. Of these patients, 55% had adenocarcinomas, of which 36.4% were adenocarcinomas of the stomach; lymphoproliferative neoplasias were the second most recorded neoplasms, accounting for 18.3%. Metastatic disease was reported in 56.7% of the patients, with an average survival time of 11.5 months after cancer diagnosis [9].

The sign of Leser-Trélat has also been associated to breast, prostate, lung, ovary, and kidney cancer and melanoma [1]. Yaniv et al. was the first to report the association of the sign with transitional cell carcinoma of the urinary-bladder, in 1994 [10]. Martínez-Morán et al., in 2007, associated the sign with Sézary syndrome and transitional cell carcinoma of the bladder [11].

In comparison to the literature, our patient was near the average age (67 years) and time of skin eruption (approximately 16 weeks) and presented pruritus in the follow-up. There was no sign of associated acanthosis nigricans on his dermatological examination. The low-stage papillary urothelial carcinoma was a rare association.

Interestingly, the link between seborrheic keratoses and bladder carcinoma has also been searched at genetic level. Fibroblast growth factor receptor 3 (FGFR3) currently appears to be the most frequently mutated oncogene in bladder cancer and implies a good prognosis [12–14]. FGFR3 mutations can also be frequently identified in seborrheic keratoses [15]. Only a few mutational hot spots account for all FGFR3 mutations detected in urothelial carcinomas and skin tumors (epidermal nevi and seborrheic keratoses) so far [16]. Therefore, a comparison of skin and urothelium—and of benign and malignant lesions from these epithelia—may provide important clues as to the genetic mechanisms involved in tumor development and progression in these tissues [17]. FGFR3 mutation was not sought in our patient investigation.

Whether the sign of Leser-Trélat is a genuine paraneoplastic phenomenon is still discussed. Critics advocate that in the elderly both seborrheic keratoses and cancer are common and their coexistence may be fortuitous [18, 19]. Lindelöf et al. have evaluated the possible association of malignant disease and the sign in 1752 consecutive cases of seborrheic keratoses and found no evidence to support the validity of the Leser-Trélat sign. Howsoever, men with seborrheic keratoses appeared to have a slightly increased risk of urinary tract neoplasia, excluding kidney (RR 2.2, IC 1.2–3.8), and women, tendency of breast cancer (RR 1.6, IC 1.1–2.2) [20]. Fink et al. counted the number and sites of seborrheic keratoses in 150 oncological patients and 150 matched controls; no association was found between these cutaneous findings and cancer [19].

Heaphy et al. suggested distinguishing between a “sign of Leser-Trélat” and a “syndrome of Leser-Trélat”: the sign would be defined as an acute efflorescence of seborrheic keratoses, may be present with or without occult malignancy, and is detectable on history and physical examination alone; furthermore, the term syndrome would then be used to describe a paraneoplastic syndrome in patients with the sign in whom an occult malignancy was identified after the appearance of the sign [8].

Many authors believe that the cumulative weight of published cases of the association of seborrheic keratoses with subsequent demonstration of malignancy shows the need for an exhaustive evaluation of all such individuals, including physical examination, radiologic and imaging studies, endoscopic studies, and other laboratory evaluations [3, 7, 8, 21]. Patients presenting the sign of Leser-Trélat should be considered to harbor an occult malignancy until further investigation rules out this hypothesis. If all investigations are negative and the cutaneous manifestation progresses, the studies should be repeated after an appropriate interval [8].

Most paraneoplastic dermatoses disappear when the primary tumor is removed and reappear in the case of recurrence or metastases of the cancer [21]. In about one-third of the patients with Leser-Trélat syndrome a parallel course of the skin disorder is observed; there is a decrease in size and number of the cutaneous lesions following appropriate surgical and/or chemotherapeutic intervention, along with the return of the seborrheic keratoses with recurrent malignancy [9]. In our patient, the number of lesions significantly declined after cancer resection.

Thereby, though a rarely observed entity, our case supports the validity of the Leser-Trélat sign and its relevance to the affected individuals, as the investigation may lead to an early diagnosis of yet low-stage cancer and then, with prompt treatment, offer a good prognosis.
